# The Complete Genome Sequence of Bacillus thuringiensis Strain ATCC 13367 Features a *cry*-Containing Chromosome

**DOI:** 10.1128/mra.01227-21

**Published:** 2022-04-04

**Authors:** P. S. Shwed, J. Crosthwait, K. Weedmark, G. Leveque

**Affiliations:** a Environmental and Radiation Health Sciences Directorate, Health Canada, Ottawa, ON, Canada; b Food Directorate, Bureau of Microbial Hazards, Health Canada, Ottawa, ON, Canada; c Canadian Centre for Computational Genomics (C3G), McGill University, Montréal, QC, Canada; University of Maryland School of Medicine

## Abstract

We report the high-quality closed genome sequence of Bacillus thuringiensis ATCC 13367, which is used as a bioinsecticide. The genome features a 328-kbp plasmid and a 5.8-Mbp chromosome. The genome is atypical for the species since it lacks a plasmid pesticidal crystal protein (*cry*) gene and instead features a chromosomal *cry* gene like that of B. thuringiensis HER1410.

## ANNOUNCEMENT

Bacillus thuringiensis is a member of the Bacillus cereus *senso lato* group and is industrially significant as a bioinsecticide. In this study, the complete sequence of B. thuringiensis Berliner ATCC 13367, which is in commercial use, was determined as part of risk assessment.

B. thuringiensis ATCC 13367 was obtained from the ATCC and is reported as a moth isolate. A single colony from brain heart infusion agar (BHI) was inoculated into 2 mL of BHI medium at 30°C with shaking at 220 rpm for 24 h. DNA was extracted using the MasterPure Gram-positive DNA purification kit (Lucigen Corporation). DNA was treated with RNase A (2 μL at 10 mg/mL for 30 min at 37°C) and purified using solid-phase reversible immobilization selection (1). Short-read genome sequences were collected on the MiSeq platform (v3 chemistry) using the 2 × 300-bp paired-end read protocol and NexteraXT library preps according to the manufacturer’s instructions (Illumina Inc.). The number of raw Illumina reads was 328,188.

Long-read genome sequences were collected on a PacBio Sequel II instrument from a large insert (>10-kb) genomic library that was constructed using the SMRTbell Express template prep kit (version 2.0; PacBio, Menlo Park, CA). All software was used with default parameters unless otherwise specified. Raw read error correction, trimming, and assembly were carried out with the Canu assembler (version 2.1.1) using default settings (2). Circlator (version 1.5.5) was used to trim any overlapping contig ends to produce circular molecules and rotated to the *dnaA* gene for the chromosome, or the middle of the plasmid contig (3). The PacBio total read number was 2,651,962, with an average read length of 8,387 bp and a total raw length of 22.2 Gbp. The raw Illumina reads were used to polish the PacBio assembly using Pilon (version 1.23) (4).

The NCBI Prokaryotic Genome Annotation Pipeline (version 4.13) (5) was used for gene annotation. B. cereus group virulence genes were detected using BTyper (version 2.3.2) (6). Insecticidal-toxin parasporal crystal *cry* genes were detected using BTyper3 (version 3.0.2) (7). Genomic islands were predicted using IslandViewer 4 (8). Genome similarity was determined by average nucleotide identity (ANI) using the OrthoANIu algorithm (9).

The closed B. thuringiensis ATCC 13367 genome contains a single megaplasmid, pBt13367-1 (328,200 bp; GC content of 32.3%), that lacks a *cry* gene and carries a partial hemolysin Bl operon (*hblC-hblD*). However, the circular chromosome (5,758,378 bp; GC content of 35.4%) features a single *cry* gene, *cry1Ba4*, at bp 16935 in a 17.4-kb genomic island (8), close to the origin, the same as in the B. thuringiensis HER1410 genome (5.59 Mbp; GC content of 39.4%) (10). The identified chromosomal B. cereus toxin genes included the gene for the diarrheal syndrome enterotoxin cytolysin K (*cytK*) and the complete operons for the nonhemolytic enterotoxin genes *nheABC* and hemolysin Bl (*hblABCD*). The genome of B. thuringiensis HER1410 is the only other known genome to feature a chromosomal *cry* gene, and the derived OrthoANIu value of 99.95% reflects the close relationship between it and B. thuringiensis ATCC 13367.

### Data availability.

The complete genome sequences of B. thuringiensis ATCC 13367 were deposited in GenBank under the accession numbers CP074713 for the plasmid sequence and CP074714 for the chromosome sequence. The raw sequence data were deposited in the SRA database under the numbers SRX10593864 (Illumina) and SRX10252734 (PacBio).

## References

[1] Hosomichi K, Mitsunaga S, Nagasaki H, Inoue I. 2014. A Bead-based Normalization for Uniform Sequencing depth (BeNUS) protocol for multi-samples sequencing exemplified by HLA-B. BMC Genomics 15:645. doi:10.1186/1471-2164-15-645. 25087472 PMC4133082

[2] Koren S, Walenz BP, Berlin K, Miller JR, Bergman NH, Phillippy AM. 2017. Canu: scalable and accurate long-read assembly via adaptive *k*-mer weighting and repeat separation. Genome Res 27:722–736. doi:10.1101/gr.215087.116. 28298431 PMC5411767

[3] Hunt M, Silva ND, Otto TD, Parkhill J, Keane JA, Harris SR. 2015. Circlator: automated circularization of genome assemblies using long sequencing reads. Genome Biol 16:294. doi:10.1186/s13059-015-0849-0. 26714481 PMC4699355

[4] Walker BJ, Abeel T, Shea T, Priest M, Abouelliel A, Sakthikumar S, Cuomo CA, Zeng Q, Wortman J, Young SK, Earl AM. 2014. Pilon: an integrated tool for comprehensive microbial variant detection and genome assembly improvement. PLoS One 9:e112963. doi:10.1371/journal.pone.0112963. 25409509 PMC4237348

[5] Tatusova T, DiCuccio M, Badretdin A, Chetvernin V, Nawrocki EP, Zaslavsky L, Lomsadze A, Pruitt KD, Borodovsky M, Ostell J. 2016. NCBI Prokaryotic Genome Annotation Pipeline. Nucleic Acids Res 44:6614–6624. doi:10.1093/nar/gkw569. 27342282 PMC5001611

[6] Carroll LM, Kovac J, Miller RA, Wiedmann M. 2017. Rapid, high-throughput identification of anthrax-causing and emetic Bacillus cereus group genome assemblies via BTyper, a computational tool for virulence-based classification of Bacillus cereus group isolates by using nucleotide sequencing data. Appl Environ Microbiol 83:e01096-17. doi:10.1128/AEM.01096-17. 28625989 PMC5561296

[7] Carroll LM, Wiedmann M, Kovac J. 2020. Proposal of a taxonomic nomenclature for the Bacillus cereus group which reconciles genomic definitions of bacterial species with clinical and industrial phenotypes. mBio 11:e00034-20. doi:10.1128/mBio.00034-20. 32098810 PMC7042689

[8] Bertelli C, Laird MR, Williams KP, Lau BY, Hoad G, Winsor GL, Brinkman FSL, Simon Fraser University Research Computing Group. 2017. IslandViewer 4: expanded prediction of genomic islands for larger-scale datasets. Nucleic Acids Res 45:W30–W35. doi:10.1093/nar/gkx343. 28472413 PMC5570257

[9] Yoon S-H, Ha S-M, Lim J, Kwon S, Chun J. 2017. A large-scale evaluation of algorithms to calculate average nucleotide identity. Antonie Van Leeuwenhoek Int J Gen Mol Microbiol 110:1281–1286. doi:10.1007/s10482-017-0844-4. 28204908

[10] Lechuga A, Lood C, Salas M, van Noort V, Lavigne R, Redrejo-Rodríguez M. 2020. Completed genomic sequence of bacillus thuringiensis HER1410 reveals a cry-containing chromosome, two megaplasmids, and an integrative plasmidial prophage. G3 (Bethesda) 10:2927–2939. doi:10.1534/g3.120.401361. 32690586 PMC7466992

